# The Injectable-Only Contraceptive Medroxyprogesterone Acetate, Unlike Norethisterone Acetate and Progesterone, Regulates Inflammatory Genes in Endocervical Cells via the Glucocorticoid Receptor

**DOI:** 10.1371/journal.pone.0096497

**Published:** 2014-05-19

**Authors:** Yashini Govender, Chanel Avenant, Nicolette J. D. Verhoog, Roslyn M. Ray, Nicholas J. Grantham, Donita Africander, Janet P. Hapgood

**Affiliations:** 1 Department of Molecular and Cell Biology, University of Cape Town, Rondebosch, Western Province, South Africa; 2 Department of Biochemistry, Stellenbosch University, Stellenbosch, Western Province, South Africa; St. Jude Children’s Research Hospital, United States of America

## Abstract

Clinical studies suggest that the injectable contraceptive medroxyprogesterone acetate (MPA) increases susceptibility to infections such as HIV-1, unlike the injectable contraceptive norethisterone enanthate (NET-EN). We investigated the differential effects, molecular mechanism of action and steroid receptor involvement in gene expression by MPA as compared to NET and progesterone (P4) in the End1/E6E7 cell line model for the endocervical epithelium, a key point of entry for pathogens in the female genital mucosa. MPA, unlike NET-acetate (NET-A) and P4, increases mRNA expression of the anti-inflammatory GILZ and IκBα genes. Similarly, MPA unlike NET-A, decreases mRNA expression of the pro-inflammatory IL-6, IL-8 and RANTES genes, and IL-6 and IL-8 protein levels. The predominant steroid receptor expressed in the End1/E6E7 and primary endocervical epithelial cells is the glucocorticoid receptor (GR), and GR knockdown experiments show that the anti-inflammatory effects of MPA are mediated by the GR. Chromatin-immunoprecipitation results suggest that MPA, unlike NET-A and P4, represses pro-inflammatory cytokine gene expression in cervical epithelial cells via a mechanism involving recruitment of the GR to cytokine gene promoters, like the GR agonist dexamethasone. This is at least in part consistent with direct effects on transcription, without a requirement for new protein synthesis. Dose response analysis shows that MPA has a potency of ∼24 nM for transactivation of the anti-inflammatory GILZ gene and ∼4–20 nM for repression of the pro-inflammatory genes, suggesting that these effects are likely to be relevant at injectable contraceptive doses of MPA. These findings suggest that in the context of the genital mucosa, these GR-mediated glucocorticoid-like effects of MPA in cervical epithelial cells are likely to play a critical role in discriminating between the effects on inflammation caused by different progestins and P4 and hence susceptibility to genital infections, given the predominant expression of the GR in primary endocervical epithelial cells.

## Introduction

A central issue in women’s health in developing countries is choice of contraceptive with minimal effects on susceptibility to infectious diseases, in particular to human immunodeficiency virus (HIV)-1 acquisition via the female reproductive tract (FRT). Epithelial cells lining the FRT are the first line of defence against pathogens and serve not only as a physical barrier but also express a wide variety of immune mediators aiding in both innate and adaptive immunity [1]–[3]. Interleukin (IL)-6, IL-8 and regulated-upon-activation-normal-T-cell-expressed-and-secreted (RANTES) are expressed in both primary and immortalised vaginal and cervical epithelial cells [4]–[6]. In particular, the simple columnar epithelial cells of the endocervix constitutively express IL-6, IL-8, and RANTES genes [5], with the endocervical cells being more active in cytokine secretion than the ectocervical cells [7], [8]. Pathogens such as herpes simplex virus (HSV), human papillomavirus (HPV), and HIV have been shown to infect epithelial cells of the FRT and the process is affected by treatment with hormones such as progesterone (P4) [9], [10].

Several reports suggest that endogenous steroid hormone levels and synthetic progestins used in contraception, influence susceptibility and disease predisposition to many genital tract infections (reviewed in [2], [11]). Treatment of animals and humans with P4 or synthetic progestins has been reported to increase susceptibility to viral and bacterial infections [12]–[16]. Consistent with these findings, the progestin medroxyprogesterone acetate (MPA) is used as an immuno-compromising agent to induce viral infectivity in mice [17]. Furthermore, a prospective cohort study reported that injectable contraceptive users are more susceptible to both chlamydia and gonococcal infections than oral contraceptive users [15]. MPA, administered for contraception as Depo-MPA (DMPA) or Depo-Provera, is a 150 mg three-monthly intramuscular injection used by millions of women worldwide, particularly in Sub-Saharan Africa with high HIV-1 incidence and prevalence [18], [19]. Norethisterone enanthate (NET-EN) is a 200 mg two-monthly injectable with less widespread use than MPA, although its usage is high in some regions of South Africa [20]. In most studies the adjusted hazard ratio for HIV-1 acquisition by DMPA, or injectable contraceptives users where the majority of women are on MPA, is higher than that associated with no contraception or oral contraception [18], [21]–[30]. While only a few studies have investigated the risks associated with the use of injectable NET-EN on HIV-1 acquisition, none have shown a significant association with HIV-1 acquisition [18], [27], [30]. In addition, increases in both HIV-1 and HSV shedding have been reported with MPA [31], [32], as well as the presence of more viral variants and higher viral loads in DMPA users infected with HIV than non-users [33]. The mechanisms whereby endogenous P4 and synthetic progestins affect pathogen entry and transmission in the FRT are not well understood, but may involve modulation of the immune response both at the systemic level and at the genital mucosa. Understanding the relative effects of MPA vs NET on genital mucosal immune function is extremely important for choice of contraception, especially for developing countries where injectable contraception usage is high. For example, at the Kwazulu-Natal site in South Africa for the CAPRISA microbicide trial, about 80% of the women investigated were on injectable progestin-only (DMPA or NET-EN), as compared to 15% on oral contraceptives [34]. HIV prevalence among young women in the general population in southern Africa is highest (about 25%) in the 20–24 age group and the ratio of the prevalence of HIV infection among women relative to men shows that these women are approximately 3.3 times more likely to be infected with HIV than young men in this region [35].

Clinical research on the effects of contraceptives on HIV-1 acquisition, transmission and disease progression has been hampered by a lack of understanding of the molecular mechanisms of action of the progestin components of contraceptives and a lack of appreciation of the differences between progestins, which cannot be considered to act as a single class of compounds regarding their side-effect profiles [36]–[38]. Although MPA and NET elicit similar progestational effects to P4 [39], [40], differences in biological effects mediated via steroid receptors other than the progesterone receptor (PR) could be expected [36]–[38], and have been demonstrated for the GR [41]. Synthetic progestins were designed to mimic the actions of the natural ligand P4 but with better bioavailability [38]. Both NET-EN and NET-A are metabolised to the active molecule NET, as well as other metabolites, unlike MPA, which is itself the active compound [42]. Progestins were also designed to be potent, high affinity PR agonists. However, many progestins bind to other members of the steroid receptor family, including the GR, the androgen receptor (AR) and mineralocorticoid receptor (MR) [43], thereby exhibiting off-target effects via these receptors [37], [38]. It has been shown that MPA has a higher relative binding affinity compared to NET-A and P4 for the human GR (relative binding affinity % of 79.1, 0.88 and 5.57 for MPA, NET-A and P4, respectively) [41] and unlike NET-A and P4, acts as a potent partial to full GR agonist for both transactivation and transrepression [41], [44]. The GR, a ligand activated steroid receptor, has potent anti-inflammatory and immunosuppressive activity [45]. Consistent with this idea, we have previously shown that MPA, at doses in the range found in serum of contraceptive users, represses expression of mRNA and protein levels of the pro-inflammatory cytokine IL-6 and the chemokine IL-8, in mouse fibroblast cells, most likely via the GR [46]. Similarly, Bamberger *et al.* showed that MPA represses IL-2, IL-1, and IL-6 protein expression in normal human lymphocytes, most likely via the GR [47]. Thus it is possible that MPA used as contraceptive modulates immune function and inflammation, and hence responses to pathogens, by changes in cytokine gene expression, particularly in the genital mucosa. A key question that remains to be investigated is what the effect is of different synthetic progestins as compared to P4 on cytokine gene expression and immune function in the FRT. These are likely to vary since we have previously shown that MPA, compared to NET-A and P4, elicit very different effects on IL-8 promoter expression in HEK293 cells, mediated via the GR [41], as well as exhibit differential effects in several steps of the GR pathway [44]. In support of an immunosuppressive role of MPA in increasing HIV-1 pathogenesis, MPA was recently shown to have immunosuppressive effects on key regulators of cellular and humoral immunity and increased HIV-1 replication in activated peripheral blood mononuclear cells (PBMCs) *ex vivo* [48], [49]. The Hel laboratory also showed that women using DMPA displayed lower levels of IFNα in plasma and genital secretions compared to controls with no hormonal contraception, consistent with an immunosuppressive effect of DMPA *in vivo* [48], [49]. A possible mechanism for differential effects of progestins and P4 on HIV-1 acquisition may include differential effects on inflammation in the FRT. However the direct effects of MPA, as compared to NET and P4, on expression of inflammatory markers in endocervical cells, the prime site for HIV-1 acquisition, have not been previously investigated. Using a human immortalised endocervical (End1/E6E7) epithelial cell line [50] as a model for the mucosal surface of the endocervix, as well as the HeLa cervical cell line, the present study aimed to determine the relative effects, molecular mechanisms and steroid receptor involvement of MPA, P4 and NET-A in expression of key inflammatory response genes.

## Materials and Methods

### Antibodies and Compounds

The following primary antibodies were obtained from Santa Cruz Biotechnology Inc., USA; GR(H-300): sc-8992, PR(C-20) (which detects PRA and B isoforms): sc-539, AR(441): sc-7305, GAPDH(0411): sc-47724, MR(MCR, H300): sc-11412, ERα(MC-20): sc-542. The flotillin-1 (610820) antibody was purchased from BD Transduction Laboratories (USA). The following secondary antibodies were obtained from Santa Cruz Biotechnology Inc., USA; anti-mouse: sc-2005, anti-goat: sc-2350 (used as IgG for the ChIP assay) and anti-rabbit: sc-2313. The ligands dexamethasone (DEX), MPA, P4, NET-A, NET, aldosterone (ALD) and mibolerone (MIB) were obtained from Sigma-Aldrich (South Africa). Human tumour necrosis factor α (TNFα) was obtained from Celtic Diagnostics (South Africa). Protease inhibitor cocktail tablets (EDTA-free) (cat #04693159001) were obtained from Roche (South Africa). Cycloheximide (CHX) was purchased from Sigma-Aldrich (South Africa).

### Cell Culture

Human epithelial cervical cancer cells (HeLa) purchased from America Type Culture Collection (ATCC, USA) were cultured in 75 cm^2^ flasks (Greiner Bio-one International, Austria) in Dulbecco’s modified Eagle’s medium (DMEM) (Sigma-Aldrich, South Africa) supplemented with 10% (v/v) foetal bovine serum (Highveld Biological, South Africa) 100 IU/mL penicillin and 100 µg/mL streptomycin (Gibco, Invitrogen, UK). End1/E6E7 (human endocervical cells immortalized with human papillomavirus E6/E7 [54] were obtained from Dr Fichorova, OB/GYN Department, Brigham & Women’s Hospital, Boston, USA. The End1/E6E7 cells were cultured in 75 cm^2^ flasks (Greiner Bio-one International, Austria) in keratinocyte serum-free medium (ker-sfm; Sigma-Aldrich, South Africa) supplemented with keratinocyte growth supplement, 100 U/ml penicillin and 100 µg/ml streptomycin (Gibco, Invitrogen, UK). All cells were maintained at 37°C in a 5% CO_2_ incubator. Cells were passaged with 0.25% trypsin/0.1% EDTA in PBS (Highveld Biological, South Africa). Trypsinization was terminated with neutralization medium [DMEM (Sigma-Aldrich, South Africa), 10% (v/v) calf serum (Highveld Biological, South Africa), 100 U/ml penicillin and 100 µg/ml streptomycin (Gibco, Invitrogen, UK)]. The cell lines were regularly tested for mycoplasma infection by means of Hoechst staining [55], and only mycoplasma-negative cells were used in experiments.

### Plasmids

pcDNA3 (empty vector) plasmid was obtained from Invitrogen, while the pcDNA3-hGR (GR) plasmid was a gift from Prof. D.W. Ray (Centre for Molecular Medicine, School of Clinical and Laboratory Sciences, University of Manchester, UK [56]. pMT-PR-B (PR) was obtained from Prof. S. Okret (Karolinska Institute, Sweden) [51]. pRS-hMR (MR) expression plasmid was obtained from Prof. R.M. Evans (University of California, USA) [52]. pSV-hAR (AR) was a kind gift from Prof.F. Classens (Catholic University of Leuven, Belgium) [53]. pSG5-hER (ER) was obtained from Prof.F. Gannon (EMBL, Germany) [54].

### RNA Isolation and Quantitative Real Time PCR (qRT-PCR)

Total RNA was isolated from cells using Tri-reagent (Sigma-Aldrich, South Africa) according to the manufacturer’s instructions, and RNA (500 ng) was reverse transcribed using the Transcriptor First Strand cDNA synthesis kit (Roche Applied Science, South Africa) according to the manufacturer’s instructions. RT-PCR was performed using the Rotor-gene, RG-3000A (Corbett Research, South Africa) according to the manufacturer’s instructions using the Sensi-Mix SYBR Green I system (Celtic Diagnostics, South Africa). The specific primer sets used were as follows; for GILZ (cat #QT00091035, Qiagen, South Africa), for IκBα, 5′-ACTCGTTCCTGCACTTGGCC-3′ (forward primer) and 5′-TGCTCACAGGCAAGGTGTAG-3′ (reverse primer), for IL-6, 5′-TCTCCACAAGCGCCTTCG-3′ (forward primer) and 5′-CTCAGGGCTGAGATGCCG-3′ (reverse primer), for IL-8, 5′-TGCCAAGGAGTGCTAAAG-3′ (forward primer) and 5′-CTCCACAACCCTCTGCAC-3′ (reverse primer), for RANTES 5′-TACCATGAAGGTCTCCGC-3′ (forward primer) and 5′-GACAAAGACGACTGCTGG-3′ (reverse primer), for GAPDH 5′-TGAACGGGAAGCTCACTGG-3′ (forward primer) and 5′-CCACCACCCTGTTGCTGTA-3′ (reverse primer). Relative transcript levels were calculated with the method described by Pfaffl*et al* 2001 and were normalized to relative GAPDH transcript levels [55].

### Western Blotting

For the steroid receptor controls, COS-1 cells were seeded into 12-well plates (Greiner bio-one, Cellstar, Austria) at a density of 25×10^4^ cells/well. The next day the cells were transfected with 1 µg/well of empty vector, GR, AR or PR and 2 µg/well of MR or ER using FuGENE 6 (Roche Diagnostics, South Africa). After 24 hrs, the cells were washed once with PBS and lysed with 50 µl 2X SDS sample buffer (5 X SDS sample buffer: 100 mM TRIS-HCL pH 6.8, 5% (w/v) SDS, 20% (v/v) glycerol, 2% β-mercaptoethanol and 0.1% (w/v) bromophenol-blue) and boiled for 10 min at 100°C. In addition, lysates were prepared from End1/E6E7 and HeLa cells seeded into 12-well plates at a density of 35×10^4^ cells/well and 15×10^4^ cells/well, respectively. Equivalent amounts of protein were loaded on either a 6% or 8% SDS-PAGE before being transferred onto a Hybond-ECL nitrocellulose membrane (Amersham, South Africa) using the Mini Protean III blotting system (Bio-Rad, South Africa). Blots were probed for anti-GR (1∶4000), anti-PR (1∶1000), anti-AR (1∶1000), anti-ER (1∶500), anti-MR (1∶1000), anti-GAPDH (1∶20 000) at 4°C overnight. Blots were washed 3 times with TBS containing 0.1% Tween for 5 mins each and subsequently incubated with horseradish peroxidise (HRP)-conjugated goat anti-rabbit (1∶10000) or goat anti-mouse (1∶5000) secondary at room temperature for 1 hr. Protein detection was performed using ECL substrate (Thermo Scientific, South Africa) with visualization on X-ray hyperfilm (Amersham, South Africa). Bands on the X-ray film were quantified using AlphaEaseFC software version 3.1.2 (Alpha Innotech Corporation).

### GR Knockdown by Small Interference RNA (siRNA)

GR knockdown was performed as previously described [56], but briefly End1/E6E7 cells were seeded in 12-well plates at a density of 35×10^4^ cells/well and incubated for 24 hrs. Thereafter, the cells were transfected with 10 nM validated GR HS_NR3C1_5 (cat #SI02654757) (Qiagen, South Africa) siRNA directed against the human GR or validated non-silencing scrambled sequence control (NSC) siRNA (cat #1027310) (Qiagen, South Africa) using HiPerfect transfection reagent (Qiagen, South Africa) as per the manufacturer’s instructions. Cells were incubated for 48 hrs before being treated for 24 hrs with 100 nM test compounds. RNA was then harvested and mRNA levels were analysed by qRT-PCR, as described above. To verify the protein knockdown, cells were transfected in parallel and analysed by Western blotting as described above.

### Luminex

Supernatants were collected from the siRNA experiments prior to cell harvest. Thereafter, cytokine protein levels were measured using a luminex assay kit according to the manufacturer’s protocol (Bio-rad, South Africa).

### Chromatin Immunoprecipitation (ChIP) Assay

ChIP was performed as described in Verhoog *et al* 2011 with modifications [56]. HeLa cells were plated at 3×10^6^ cells per dish in 15 cm dishes and grown for 24 hrs in full DMEM, before changing to phenol red-free DMEM (Sigma-Aldrich, South Africa) for an additional 24 hrs. Thereafter, the cells were incubated with serum-free, phenol-free DMEM for 2 hrs, before treatment with 100 nM DEX, MPA, P4 and NET-A for 1 hr. Cells were crosslinked for 10 min at 37°C with1% formaldehyde and the reaction was stopped with 0.1 mM glycine for 5 min, shaking at room temperature. Cells were scraped in PBS, pelleted by centrifugation and resuspended in 500 µl nuclear lysis buffer (1% SDS, 50 mMTRIS-HCL, pH 8.0, 10 mM EDTA, 1x protease inhibitor cocktail), before sonication. For immunoprecipitation, 100 µg DNA was pre-cleared with protein A/G agarose beads (sc-2003, Santa Cruz Biotechnology, USA) for 1 hr, rotating at 4°C, before incubating with 2 µg anti-GR (H300) (Santa Cruz Biotechnology, USA) or 2 µg anti-goat (Santa Cruz Biotechnology, USA), as IgG control, overnight on a rotator at 4°C. The following day, the complex was precipitated with protein A/G agarose beads for 6 hrs at 4°C, before being washed sequentially with 1 ml each of wash buffer I, II and III [57], followed by three washes with 1 ml TE buffer [10 mMTris pH 8, 0.1 mM EDTA]. Proteins were eluted from the beads by addition of 300 µl elution buffer (1% SDS, 100 nM NaHCO_3_), before the addition of 300 nM NaCl and incubation at 65°C overnight to reverse crosslinks. The following day 15 nM EDTA, 125 nM TRIS-HCL pH 6.5 and 20 µg proteinase K (Roche, South Africa) were added and samples were then incubated at 45°C for 1 hr. DNA was purified using PCR cleanup columns (Qiagen, South Africa). Real time qRT-PCR was performed on a Corbett Rotorgene, using the Sensimix (Quantace, South Africa), which measures SYBR Green fluorescence. ChIP primers used: for IL-6 5′-TCTACAACAGCCGCTCACAG-3′ (forward primer) and 5′- AGCGTTCCAGTTAATTTGTATTTGT-3′ (reverse primer), for IL-8 5′-GGGCCATCAGTTGCAAAT-3′ (forward primer) and 5′-TTCCTTCCGGTG GTTTCTTC-3′ (reverse primer).

### Primary Cervical Epithelial Cells

Primary cervical epithelial cells (VEN-100) were bought from Mat Tek Corporation (USA). Delivery time was 5 days. Upon arrival, the cells were incubated overnight in VEN-100-MM medium (Mat Tek Corporation, USA) at 37°C in a 5% CO2 incubator. The following day cell viability was determined using the Thiazolyl Blue Tetrazolium Bromide (MTT) cell determination kit (cat #CGD1, Sigma-Aldrich, South Africa) according to the manufacturer’s instructions [58]. At this time, some cells were washed with PBS and either lysed with a N-[Tris(hydroxymethyl)- methyl]-3-aminopropanesulfonic acid (TAPS) buffer (0.1 M TAPS, pH 9.5) on ice (to perform Western blotting as above) or with TRIzol® (for RNA isolation, cDNA synthesis and qRT-PCR as described above). Having established the viability of the cells, the majority of the VEN-100s were incubated in VEN-100-ASY-HCF hydrocortisone free assay medium (Mat Tek Corporation, USA) and 100 nM test compound for 48 hours, before performing an additional MTT assay.

## Results

### MPA, but not NET-A, Acts like a Full to Partial GR Agonist for Upregulation of anti-Inflammatory and Downregulation of Pro-inflammatory mRNAs

**Figure 1:**
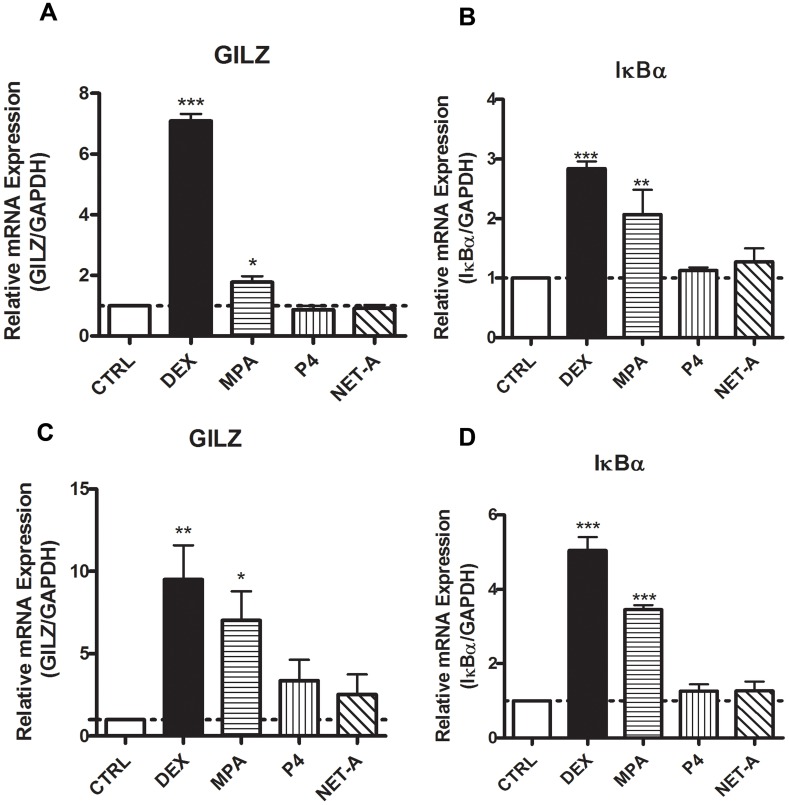
MPA, but not NET-A or P4, acts like a partial GR agonist for upregulation of anti-inflammatory mRNAs. (**A and B**) End1/E6E7 cells were treated for 24 hrs with 100 nM DEX, MPA, P4, NET-A or vehicle (ethanol) (CTRL). (**C and D**) HeLa cells were treated for 24 hrs with 100 nM DEX, 1 µM MPA, 10 µM P4, 10 µM NET-A or vehicle (ethanol) (CTRL). Thereafter the cells were harvested; total RNA was isolated and reverse-transcribed. Relative GILZ and IκBα gene expression was measured by real-time qRT-PCR and normalised to GAPDH mRNA expression. In addition, relative gene expression was normalized to basal activity (CTRL) in order to obtain relative fold expression. Graphs represent pooled results of at least three independent experiments and are plotted as mean ±SEM. Statistical analysis was carried out using GraphPad Prism software (version 5) using a one-way ANOVA with Dunnett post-test. Statistical significance is denoted by *, ** or *** to indicate P<0.05, P<0.001 or P<0.0001, respectively.

**Figure 2:**
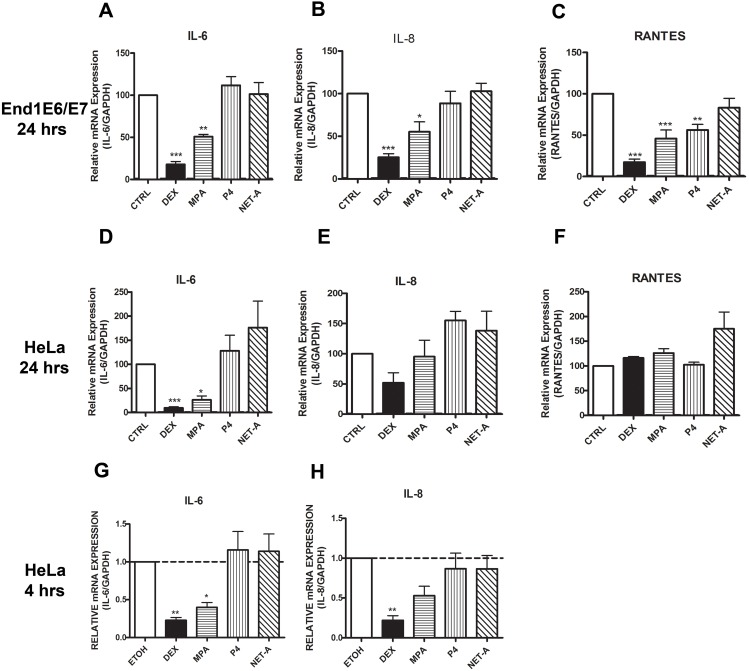
MPA, but not NET-A, acts like a full/partial GR agonist for repression of pro-inflammatory mRNAs. **(A–C)** End1/E6E7 cells were treated for 24 hrs with 100 nM DEX, MPA, P4, NET-A or vehicle (ethanol) (CTRL). (**D–H**) HeLa cells were treated for 24 hrs (**D–F**) or 4 hrs (**G–H**) with 100 nM DEX, 1 µM MPA, 10 µM P4, 10 µM NET-A or vehicle (ethanol) (CTRL). Thereafter the cells were harvested, total RNA was isolated and reverse-transcribed. Relative IL-6, IL-8 and RANTES gene expression was measured by real-time qRT-PCR and normalised to GAPDH mRNA expression. In addition, relative gene expression was normalized to basal activity (CTRL) in order to obtain relative fold expression. Graphs represent pooled results of at least three independent experiments and are plotted as mean ± SEM. Statistical analysis was carried out using GraphPad Prism software (version 5) using a one-way ANOVA with Dunnett post-test. Statistical significance is denoted by *, ** or *** to indicate P<0.05, P<0.001 or P<0.0001, respectively.

We investigated the effects of the synthetic progestins on the expression of GR regulated inflammatory genes in the End1/E6E7 endocervical epithelial cell line as well as the HeLa cervical epithelial cell line. These cell lines were chosen as the model systems for this study due to the ability to perform mechanistic studies using current methodology. Furthermore, the End1/E6E7 cell line displays similar morphological and immunocytochemical properties to those of primary endocervical epithelial cells [50]. The genes investigated were chosen based on their established mechanism of regulation via the GR, and their constitutive expression in endocervical epithelial cells [7]. GILZ and IκBα are anti-inflammatory genes that are upregulated by glucocorticoids (GCs) such as DEX, while IL-6, IL-8 and RANTES are pro-inflammatory genes that are downregulated by DEX [59], [60]. The GILZ and IκBα genes contain multiple glucocorticoid response elements (GREs) and are commonly referred to as GR transactivation genes [61], [62]. The IL-6 and IL-8 gene promoters have binding sites for transcription factors that include activator protein-1 (AP-1) and nuclear factor κB (NFκB) [63], and these genes are transrepressed by the liganded GR via tethering mechanisms [64]. Cells were treated with P4, MPA and NET-A, as well as the GR agonist DEX for 24 hrs. Thereafter, cytokine gene mRNA was measured by real time qRT-PCR. As expected the GR synthetic agonist DEX upregulated both GILZ and IκBα mRNA in both the cell lines (Figure 1). In addition, MPA upregulated GILZ and IκBα mRNA in both End1/E6E7 and HeLa cell lines (Figure 1). P4 and NET-A have no effect on the expression of GILZ or IκBα mRNA in either of the cell lines (Figure 1). Figure 2 A and B show that DEX and MPA, unlike NET-A and P4, repress both IL-6 and IL-8 mRNA levels, respectively, in the End1/E6E7 cell line. Interestingly, RANTES mRNA levels are repressed by DEX, MPA and P4 (Figure 2 C). The regulation of IL-6 mRNA levels by the ligands in the HeLa cells (Figure 2 D) is similar to the End1/E6E7 cells (Figure 2 A), where both DEX and MPA repress IL-6 mRNA levels. Furthermore, it appears that NET-A upregulates IL-6 mRNA levels in the HeLa cells (Figure 2 D). Similar to the End1/E6E7 cell line, DEX appears to repress IL-8 mRNA levels in the HeLa cells (Figure 2 E). However, unlike the End1/E6E7 cell line, at the 24 hr time point MPA does not appear to effect IL-8 mRNA expression in the HeLa cells (Figure 2 E). Interestingly though, at a 4 hr time point both DEX and MPA repress IL-8 gene expression in the HeLa cell line (Figure 2 H). It appears that NET-A and P4 upregulate IL8 mRNA levels in the HeLa cells (Figure 2 E). In contrast to the End1/E6E7 cells, the DEX, MPA and P4 repression of RANTES mRNA levels does not occur in the HeLa cells (Figure 2 F), although this could be due to low basal levels of RANTES in HeLa cells (as indicated by real time qRT-PCR Ct values, data not shown). Interestingly, it appears that NET-A upregulates RANTES mRNA levels (Figure 2 F). Taken together, these results show that MPA acts like the GR agonist DEX in upregulating GILZ and IκBα anti-inflammatory gene and generally downregulating IL-6, IL-8 and RANTES pro-inflammatory gene mRNA levels, unlike P4 and NET-A, with some exceptions. The results also suggest cell-specific, gene-specific and temporal differences in the regulation of some of the genes in response to the ligands, such as undetectable repression of IL-8 and RANTES mRNA by MPA in HeLa cells at 24 hrs, but similar repression of IL-8 in HeLa cells at 4 hrs compared to IL-8 in End1/E6E7 cells at 24 hrs. In addition, some experiments show repression by P4 of RANTES in the End1/E6E7 cells at 24 hrs, unlike in HeLa cells. Furthermore, it appears that regulation of mRNA levels by the ligands may be time dependent (Figure 2). The experiments in Figure 1 and 2 were performed in the absence of induction of the cytokine/chemokine genes with a pro-inflammatory ligand, since these genes are constitutively expressed in cervical epithelial cells. Experiments performed in the presence of TNFα, to mimic infection, showed a similar % repression of the pro-inflammatory genes with DEX and MPA, unlike P4 and NET (Figure S1). All further experiments were performed in the absence of TNFα.

### MPA Regulation of Inflammatory Gene mRNA Levels is dose- and Time-dependent

**Figure 3:**
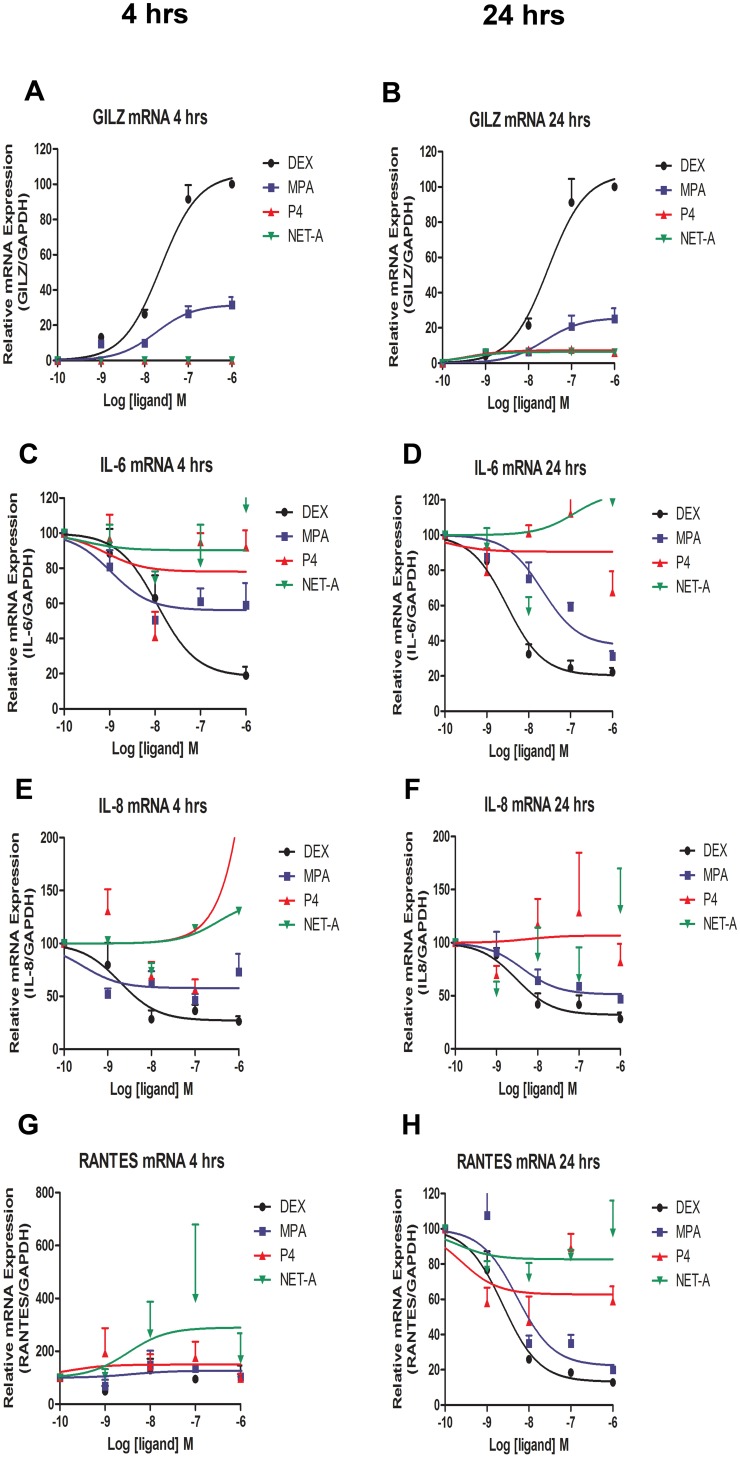
MPA-mediated regulation of inflammatory gene mRNA levels is dose- and time-dependent. End1/E6E7 cells were treated with increasing amounts (1 nM, 10 nM, 100 nM and 1 µM) of DEX, MPA, P4 or NET-A, or vehicle (ethanol) (CTRL) for 4 and 24 hrs, respectively. Thereafter, the cells were harvested, total RNA was isolated and reverse-transcribed. Relative (**A, B**) GILZ, (**C, D**) IL-6, (**E, F**) IL-8 and (**G, H**) RANTES gene expression was measured by real-time qRT-PCR and normalised to GAPDH mRNA expression. In addition, relative GILZ gene expression was normalized to 1 µM DEX set to 100% in order to obtain % partial agonist activity. Relative IL-6, IL-8 and RANTES expression was normalized to basal activity (CTRL) set to 100 in order to obtain % repression. For IL-6 and RANTES mRNA, statistically significant repression with MPA relative to control was found at 10 nM, 100 nM and 1 µM. The 1 µM data point for P4 on IL-8 4 hrs (**E**) is 231% and is not displayed due to the y-axis scale. Graphs represent pooled results of at least three independent experiments and are plotted as mean ± SEM.

Having shown that MPA acts like a GR agonist in regulating mRNA levels of inflammatory genes, it was next determined if this regulation is dose- and/or time-dependent. End1/E6E7 cells were treated with increasing concentrations of the ligands for 4 hr and 24 hrs, respectively, followed by qRT-PCR analysis. Figure 3 A and B show that both DEX and MPA increase GILZ mRNA levels in a dose-dependent manner, whileP4 and NET-A appear to have no effect on GILZ gene expression at any of the concentrations or time points. It also appears that the maximal response for MPA and DEX regulation of GILZ mRNA levels does not change between 4 and 24 hours. DEX and MPA, unlike P4 and NET-A, repress IL-6 mRNA levels in a dose-dependent mannerat both 4 hrs and 24 hrs (Figure 3 C and D). However, MPA appears to show a greater maximal repression of IL-6 mRNA levels at 24 hrs than at 4 hrs, acting like a partial agonist at 4 hrs, but a full agonist at 24 hrs. Interestingly, it appears that 1 µM NET-A may upregulate IL-6 mRNA at 24 hrs. Figure 3 E and F show that IL-8 mRNA levels are also dose-dependently repressed by DEX and MPA at both 4 hrs and 24 hrs, with the MPA dose-dependent repression of IL-8 being more robust at 24 hrs. In addition, P4 and NET-A appear to be upregulating IL-8 at 4 hrs only. No repression of RANTES mRNA is apparent at 4 hrs (Figure 3 G). However, at 24 hrs RANTES mRNA levels are repressed by DEX and MPA in a dose-dependent manner, while NET-A and P4 appear to show some partial agonist activity (10–20%) for repression at high concentrations (Figure 3 H). MPA appears to have a potency (EC_50_) of ∼24 nM for transactivation of GILZ and a potency of ∼21, 4 and 5 nM for repression of IL-6, IL-8 and RANTES mRNA, respectively at 24 hrs.

### The GR is the Predominant Steroid Receptor Protein Detected in Cervical Cell Lines and Primary Cervical Epithelial Cells

**Figure 4:**
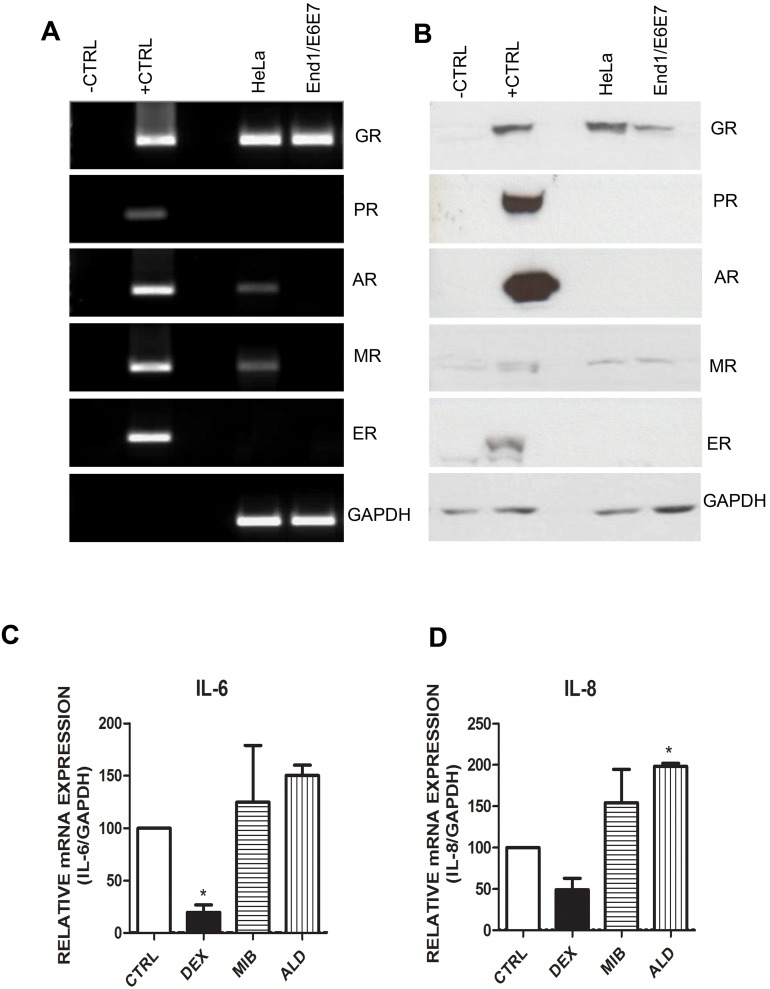
End1/E6E7 and HeLa cells only express detectable GR protein. (**A and B**) (**A**) HeLa and End1/E6E7 cells were harvested, total RNA was isolated and reverse-transcribed. Steroid receptor (SR) gene expression was measured by real time qRT-PCR. SR expression vectors (pcDNA3-hGR, pMT-PR-B, pSV-hAR, pRS-hMR and pSG5-hER) served as positive controls (+CTRL) for the GR, PR-B, MR and ER, respectively. COS-1 cells transiently transfected with pcDNA3 (empty vector) served as negative control (−CTRL). (**B**) Whole cell lysates were prepared from the HeLa and End1/E6E7 cell lines. Equal volumes of lysate were analysed by Western blotting with antibodies against specific SRs and GAPDH as loading control. **(C and D) SR agonist screen indicates that in the cervical cells the GR, but not the MR or AR repress IL-6 and IL-8 in the presence of receptor-specific agonist.** HeLa cells were treated with 100 nM DEX, 100 nM mibolerone (MIB), 10 nM aldosterone (ALD) or vehicle (ethanol) (CTRL) for 4 hrs. Total RNA was isolated and reverse-transcribed. Relative (**C**) IL-6 and (**D**) IL-8 gene expression was measured by real-time qRT-PCR and normalised to GAPDH mRNA expression. In addition, relative gene expressions were normalized to basal activity (CTRL) in order to obtain fold expression. The primers and antibody used to investigate PR levels are capable of detecting both PR-A and PR-B isoforms, however the positive protein control shown is specific for PR-B isoform only. Graphs represent pooled results of at least three independent experiments and are plotted as mean ± SEM. Statistical analysis was carried out using GraphPad Prism software (version 5) using a one-way ANOVA with Dunnett post-test. Statistical significance is denoted by * to indicate P<0.001.

Given the differential steroid receptor selectivity of MPA, NET and P4, we next investigated whether the GR, AR, PR, MR or ERα are expressed in these cell lines, with a view to determination of steroid receptor involvement in the differential gene expression responses. Cell lysates were prepared and the steroid receptor mRNA and protein levels were detected by qRT- PCR and Western blotting, respectively. The Western blot and PCR screen show that the End1/E6E7 cells express only endogenous GR mRNA and protein, respectively (Figure 4 A and B). According to the PCR screen (Figure 4 A), HeLa cells express endogenous GR, AR and MR mRNA. However the Western blot (Figure 4 B) reveals that in HeLa cells only endogenous GR protein is detectable. Although it appears that the HeLa and End1/E6E7 cells express MR protein (Figure 4 B), this is a non-specific band that also appears in the negative control. Since the End1/E6E7 cells do not express detectable MR mRNA it is highly unlikely that the cells express MR protein. However, it is possible that the HeLa cells do express low levels of the AR and MR that are beyond the detection level of the Western blots. It was therefore determined if the repression of cytokine genes in this cell line could be mediated via the AR and MR. HeLa cells were treated with the GR, AR and MR specific agonists (DEX, mibolerone and aldosterone, respectively) and cytokine gene expression was measured by qRT-PCR. IL-6 and IL-8 gene expression was measured since it was established above that RANTES is not regulated by the ligands of interest in the HeLa cells. Figure 4 C shows that only DEX represses IL-6 gene expression, while it appears that aldosterone, and possibly and mibolerone, upregulate IL-6 mRNA expression. In addition, it appears that DEX represses IL-8, while aldosterone, and possibly mibolerone, upregulate IL-8 gene expression (Figure 4 D). These results indicate that DEX- and MPA-mediated repression of the cytokine genes in HeLa cells is likely to occur via the GR. Additionally, since only GR mRNA and protein were detected in the End1/E6E7 cells, these results indicate that in both the cell lines, the DEX and MPA regulation of expression of the inflammatory genes is most likely mediated via the GR.

**Figure 5:**
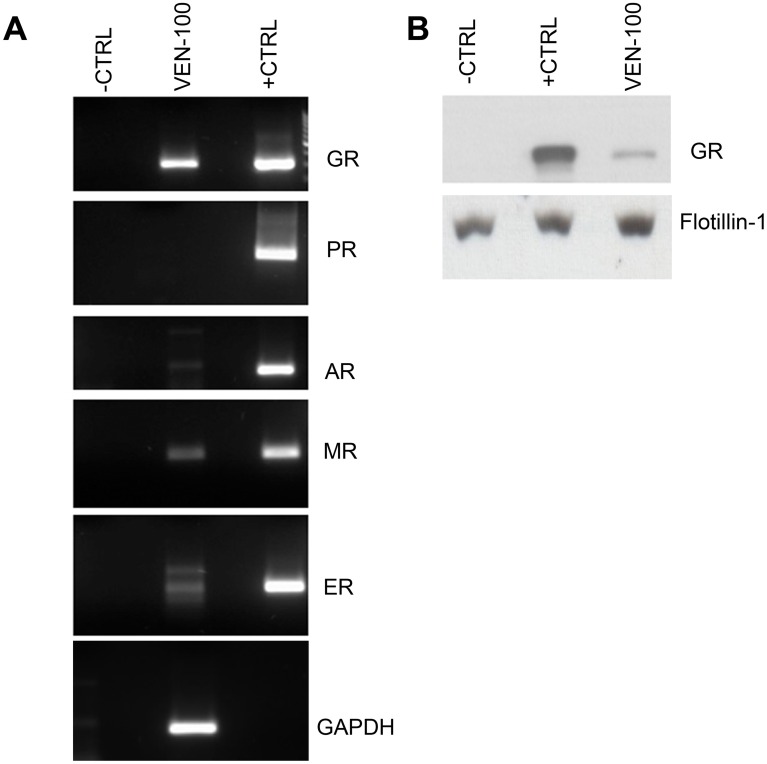
Only GR protein is detected in primary cervical epithelial cells (VEN-100). (**A**) Upon arrival the VEN-100 cells were rested overnight before being washed once with PBS and harvested with TRIzol®. Total RNA was isolated and 500 ng RNA was reverse-transcribed. Steroid receptor gene expression was measured by qRT-PCR with receptor-specific primers, followed by gel electrophoresis to confirm the PCR products. (**B**) VEN-100 cells were rested overnight before harvesting in 2X SDS sample buffer. COS-1 cells were transiently transfected with 1 µg/well pcDNA3 (empty vector) which served as negative control (−CTRL) or with 1 µg/well steroid receptor expression vectors (GR, PR-B, AR, MR and ERα) which served as positive controls (+CTRL). Twenty fourhrs later, the COS-1 cells were washed once and lysed with 2X SDS sample buffer. Equal volumes of cell lysate (VEN-100 and COS-1 ctrls) were analysed by Western blotting with antibodies specific for the GR and Flotillin-1 (loading control), respectively.

Whether the steroid receptor expression profile in the cervical epithelial cell lines mimics that of primary cervical epithelial cells is unknown. We investigated the steroid receptor content in commercially available primary endocervical cells (VEN-100; bio-engineered multilayer of primary cells) by PCR and Western blot. While we could detect GR, MR, AR and ERα mRNA (Figure 5 A), the only steroid receptor protein we detected in the primary cells was the GR (Figure 5 B). No PR mRNA or protein was detected in the cell lines or primary cells, despite positive controls showing that PR expression can be detected by these methods. Thus despite the finding that some MR, AR or ERα mRNA was detected in some of the cells lines or primary cells, the only steroid receptor protein detected in any of the models was the GR, suggesting that the GR is the predominant steroid receptor mediating responses to MPA in both the cervical epithelial cell lines and primary endocervical cells.

### Regulation of Inflammatory Gene mRNA Levels by DEX and MPA is Mediated by the GR and is Mimicked at the Protein Level for IL-6 and IL-8

**Figure 6:**
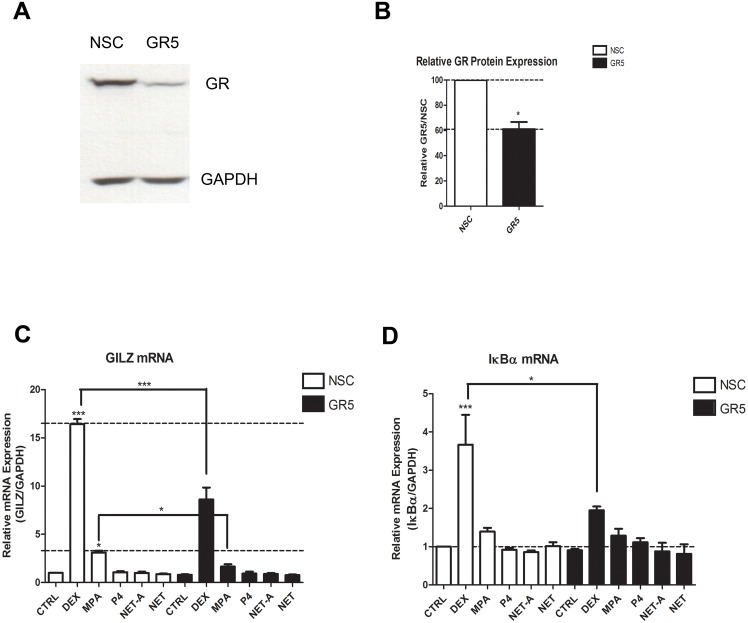
MPA- and DEX-mediated upregulation of anti-inflammatory mRNAs is mediated via the GR. End1/E6E7 cells were transfected with 10 nM GR or NSC siRNA (**A–D**) and then treated for 24 hrs with 100 nM DEX, MPA, P4, NET-A, NET or vehicle (ethanol) (CTRL). For verification of GR knockdown a representative blot is shown in (**A**). (**B**) Western blots of at least three independent experiments were quantified to determine the relative GR protein expression and is plotted as mean ± SEM. Total RNA was isolated and reverse-transcribed. Relative (**C**) GILZ and (**D**) IκBα gene expression was measured by real-time qRT-PCR and normalised to GAPDH mRNA expression. In addition, relative gene expressions were normalized to basal activity (CTRL) in order to obtain relative fold expression. Graphs in (**C**) and (**D**) represent pooled results of at least three independent experiments and are plotted as mean ± SEM. Statistical analysis was carried out using GraphPad Prism software (version 5) using a one-way ANOVA with either a Dunnett post-test, followed by a student’s t-test to compare specific conditions to each other. Statistical significance is denoted by * or *** to indicate P<0.05 or P<0.0001, respectively.

**Figure 7:**
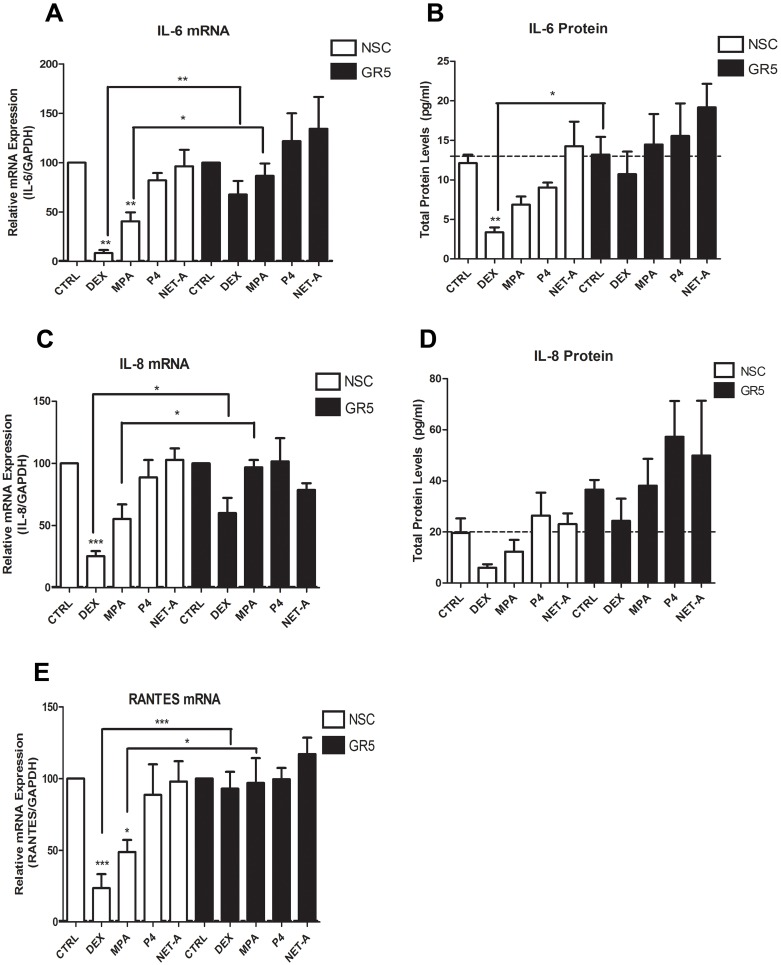
MPA- and DEX-mediated repression of pro-inflammatory cytokine gene mRNA is mediated via the GR. End1/E6E7 cells were transfected with 10 nM GR or NSC siRNA (**A–F**) and then treated for 24 hrs with 100 nM DEX, MPA, P4, NET-A or vehicle (ethanol) (CTRL). Total RNA was isolated and reverse-transcribed. Relative (**A**) IL-6 (**C**) IL-8 and (**E**) RANTES gene expression was measured by real-time qRT-PCR and normalised to GAPDH mRNA expression. In addition, relative gene expressions were normalized to basal activity (CTRL) in order to obtain fold expression. The corresponding cytokine protein levels for (**B**) IL-6 and (**D**) IL-8 were determined by Luminex of supernatants collected prior to cell harvest. Graphs represent pooled results of at least three independent experiments and are plotted as mean ± SEM. Statistical analysis was carried out using GraphPad Prism software (version 5) using a one-way ANOVA with a Dunnett post-test followed by a student’s t-test to compare specific conditions to each other. Statistical significance is denoted by * or ** to indicate P<0.05 or P<0.001, respectively.

In order to provide direct proof that the GR is involved in the regulation of the inflammatory genes in response to the synthetic progestin MPA, GR knockdown experiments were performed in the End1/E6E7 cell line. Reduction of GR protein in these cells was verified by Western blotting (Figure 6 A and 6B). As expected DEX and MPA upregulated GILZ mRNA, while P4, NET-A and NET did not (Figure 6 C). Notably, NET was included in this experiment as a control to exclude the possibility that the acetate form (NET-A) would regulate the genes differently. However, it is shown that NET-A acts similarly to NET. Both the DEX- and MPA-induced upregulation of GILZ mRNA is diminished when GR is knocked down. Figure 6 D shows that DEX upregulates IκBα mRNA levels and this induction is repressed when GR is knocked down. Here the MPA induction of IκBα is not significant, possibly due to the blunting of the response in the NSC knockdown conditions, and therefore a loss of induction is not apparent with the knockdown. Western blotting revealed that, unlike for the mRNA levels, DEX, MPA and NET-A all significantly increased total IκBα protein levels (Figure S2). Protein levels could not be determined for GILZ due to the unavailability of a suitable antibody. As expected DEX and MPA repress IL-6 mRNA levels (Figure 7 A), which is lifted when the GR is knocked down. In addition, DEX-mediated repression is also evident on IL-6 protein levels (Figure 7 B). Consistent with the mRNA data, MPA appears to repressIL-6 protein levels and the repression is lifted in the knockdown. Interestingly P4 also appears to repress IL-6 protein levels, although significance could not be established. Similarly, a significant difference is observed for both DEX and MPA responses upon GR knockdown for IL-8 mRNA levels (Figure 7 C). Figure 7 D shows that DEX and MPA also appear to repress IL-8 protein levels, while GR knockdown appears to lift this repression. RANTES mRNA levels are shown in Figure 7 E to be repressed by both DEX and MPA, but not by P4 or NET-A, in a GR-dependent manner. We were unable to detect secreted RANTES protein, possibly due to its instability in the medium (data not shown). Gene expression studies could not be performed with the primary cells since they did not maintain cell viability for the long periods of time required for the assessment (Figure S3). Taken together, these results show that DEX- and MPA-mediated regulation of the inflammatory gene mRNA levels is mediated via the GR in the endocervical cell line. This GR dependence is mimicked at the protein level for DEX and appears to also be mimicked at the protein level for MPA, for IL-6 and IL-8.

We have previously shown in COS-1 cells that the most potent GR ligands result in the most rapid GR degradation, with a good correlation shown between ligand-selective GR half-life and transactivation and transrepression efficacy [65]. Consistent with these results, we show that in the End1/E6E7 cells, MPA results in GR turnover typical for a relatively potent GR partial agonist, unlike NET-A and P4 (Figure S4).

### Inhibition of Protein Synthesis Supports a Mechanism of Direct Regulation by the GR of the Inflammatory Genes

**Figure 8:**
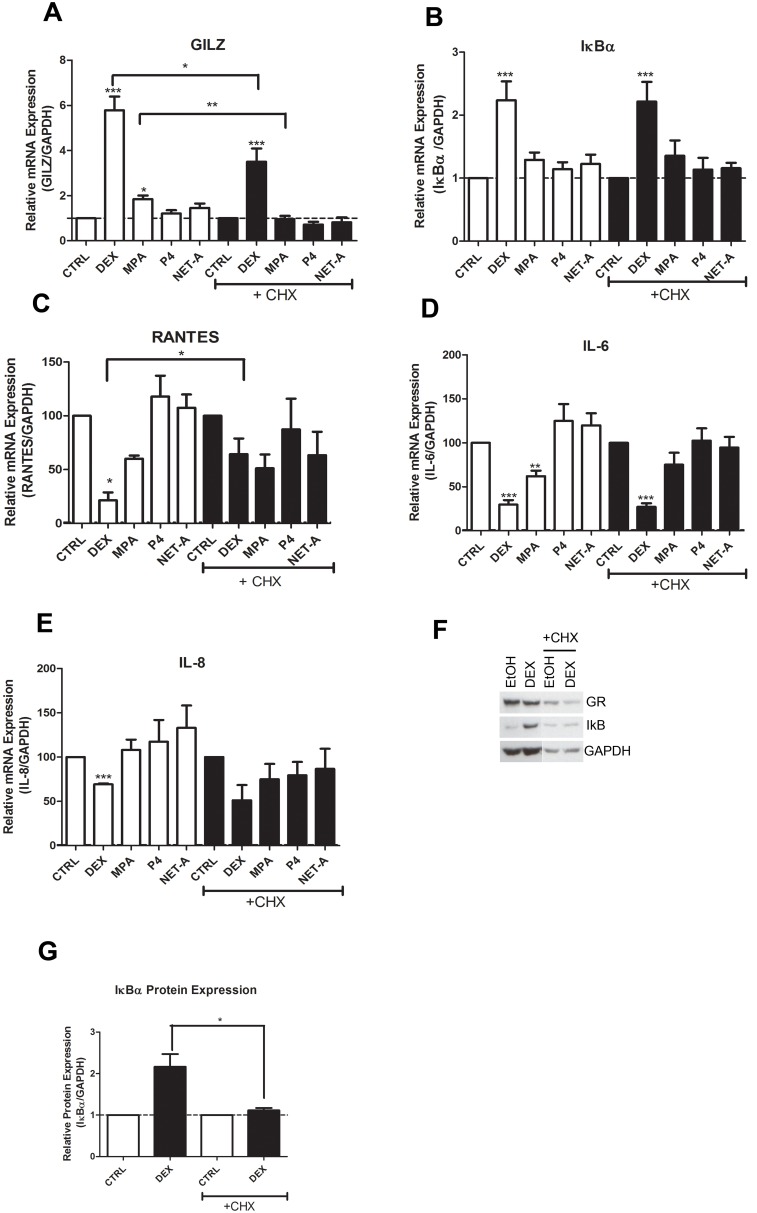
The GR at least in part directly regulates mRNA levels of the inflammatory genes. End1/E6E7 cells were pretreated with 1 µg/ml cycloheximide (CHX) then treated for 24 hrs with 100 nM DEX, MPA, P4, NET-A or vehicle (ethanol) (CTRL), in the absence or presence of CHX. Total RNA was isolated and reverse-transcribed. Relative (**A**) GILZ (**B**) IκBα, (**C**) RANTES, (**D**) IL-6 and (**E**) IL-8 gene expressions was measured by real-time qRT-PCR and normalised to GAPDH mRNA expression. In addition, relative gene expressions were normalized to basal activity (CTRL) in order to obtain relative fold expression. Graphs represent pooled results of at least three independent experiments and are plotted as mean ± SEM. To verify that the CHX inhibited *de novo* protein synthesis, End1/E6E7 cells were pretreated with CHX then treated with 100 nM DEX or vehicle (ethanol) (CTRL) for 24 hrs. (**F**) Cells were harvested and equal volumes of lysate were analysed by Western blotting with an antibody specific for IκBα and a GAPDH specific antibody as loading control. (**G**) Western blots of four independent experiments were quantified to determine the relative GR protein expression. Statistical analysis was carried out using GraphPad Prism software (version 5) using a one-way ANOVA with a Dunnett post-test followed by a student’s t-test to compare specific conditions to each other. Statistical significance is denoted by *, ** or *** to indicate P<0.05, P<0.001 or P<0.0001, respectively.

In order to investigate whether the GR is directly or indirectly involved in the regulation of these genes, cycloheximide (CHX; *de nov*o protein synthesis inhibitor) experiments were performed in the End1/E6E7 cells to determine whether the GR-mediated regulation of the mRNA levels requires new protein synthesis [66]. Figure 8 A shows that the addition of CHX only partially dampens the DEX while ablating the MPA induction of GILZ mRNA. However, the effects of all the ligands on IκBα mRNA levels were unchanged by CHX (Figure 8 B). These results suggest that upregulation of GILZ mRNA levels is only partially dependent on transactivation by the GR and it is also in part dependent on synthesis of another protein. IκBα mRNA upregulation, however, appears independent of new protein synthesis, suggesting that the mechanism predominantly involves direct transactivation by the GR of the IκBα gene. Figure 8 C shows that DEX, but not MPA-mediated repression of RANTES is partially lifted by treatment with CHX, suggesting a mechanism at least partially involving transrepression of these promoters by the GR. In contrast, both DEX- and MPA-mediated repression of IL-6 are independent of new protein synthesis, as they are not affected by CHX treatment (Figure 8 D). A similar trend is observed for DEX on the IL8 promoter (Figure 8 E), although for this gene the results for MPA were inconclusive. To confirm that the CHX inhibited *de nov*o protein synthesis, End1/E6E7 cells were pre-treated with CHX and then treated with DEX (in the presence of CHX) for 24 hrs, thereafter cell lysates were prepared and Western blotting was performed. lκBα protein levels were used as a positive control to show that the concentration of CHX used was sufficient to prevent new protein synthesis (Figure 8 F and G). In summary, we demonstrate that under conditions where CHX is shown to inhibit new protein synthesis, all the anti-inflammatory and pro-inflammatory genes investigated are at least in part regulated by direct effects of DEX without a requirement for new protein synthesis, and where this could be established, similar trends are observed for MPA.

### DEX and MPA Result in Recruitment of the GR to the Promoters of the IL-6 and IL-8 Genes

**Figure 9:**
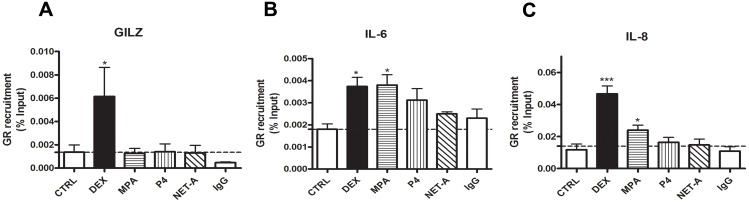
DEX and MPA recruit GR to the IL-6 and IL-8 cytokine gene promoters. HeLa cells were serum starved for 2-A or vehicle (ethanol) (CTRL). ChIP was carried out using an anti-GR antibody to immunoprecipitate endogenous GR and an anti-IgG antibody as a negative control. Real-time qRT-PCR was performed on input and immunoprecipitated DNA with primers specific for endogenous (**A**) GILZ, (**B**) IL-6 and (**C**) IL-8 promoters. GR recruitment was measured relative to input. Graphs represent pooled results of at least three independent experiments and are plotted as mean ± SEM. Statistical analysis was carried out using GraphPad Prism software (version 5) using a one-way ANOVA with Dunnett post-test. Statistical significance is denoted by * or *** to indicate P<0.05 or P<0.0001, respectively.

In order to further investigate the mechanism of transcriptional regulation of these cytokine genes via the GR, ChIP assays were performed in HeLa cells. Attempts to perform ChIP assays in the End1/E6E7 cells were unsuccessful. This may be due to high background and low sensitivity for ChIP signals in these cells. Figure 9 A shows that stimulation with DEX, but not MPA results in the recruitment of the GR to the GILZ promoter. Furthermore, both DEX and MPA stimulation resulted in significant recruitment of the GR to the IL-6 and IL-8 promoters (Figure 9 B and C). The inability to observe GR recruitment to the GILZ promoter with MPA may be because some of the effects of MPA on GILZ are not direct, as suggested by the CHX experiments. However, since we have previously shown in A549 cells that the GR is recruited to the GRE region of the GILZ promoter by both DEX and MPA [67], it is more likely that a small amount of GR is recruited by MPA, but this is below the limits of detection of the ChIP assay in these cells. In summary these results strongly support a model whereby both DEX and MPA suppress inflammation in the cervical epithelial cells by activating and thereby recruiting the GR to promoters of these genes and consequently inducing transcription of the anti-inflammatory gene GILZ, while repressing transcription of the pro-inflammatory genes IL-6 and IL-8.

## Discussion

We show for the first time that the synthetic progestins MPA and NET-A, used in contraception and hormone replacement therapy, exert differential effects on expression of mRNA levels of key pro-inflammatory and anti-inflammatory genes constitutively expressed in an endocervical epithelial cell line, as compared to P4. MPA, unlike NET-A and P4, increases mRNA expression of the anti-inflammatory genes GILZ and IκBα, in both the cervical epithelial cells lines. Interestingly, this differential regulation of IκBα mRNA is not mimicked by IκBα protein levels, suggesting that GR-mediated increase in IκBα protein levels does not play a major role in regulation of IL-6, IL-8 and RANTES genes in these cells, consistent with reports for some cells but not others [68]–[72]. MPA unlike NET-A, decreases expression of the pro-inflammatory IL-6, IL-8 and RANTES genes in the endocervical epithelial cell line, as well as IL-6 and IL-8 in the HeLa cell line. These effects are mimicked at the protein levels for IL-6 and IL-8 in the epithelial cell line. Thus MPA, unlike NET-A and P4, shows an anti-inflammatory profile in both cell lines, for most genes investigated. Furthermore, we show for the first time that the predominant steroid receptor protein detected in the endocervical epithelial cell line and in primary endocervical epithelial cells is the GR, with no detectable PR mRNA or protein. Consistent with this finding, we also demonstrate by a combination of GR knockdown and ChIPs, that MPA, unlike NET-A, represses pro-inflammatory cytokine gene expression in cervical epithelial cells via a mechanism involving recruitment of the GR to cytokine gene promoters. These results are consistent with a direct effect of the GR without a requirement for new protein synthesis, as shown by cycloheximide experiments. Our findings that DEX recruits GR to the IL-6 and IL-8 promoter regions are consistent with previous reports [56], [73], while we show here for the first time, that stimulation with MPA recruits GR to the IL-6 and IL-8 promoter regions, thereby repressing expression of these genes. These results are consistent with our hypothesis and our previously published data that MPA, unlike NET-A or P4, acts like a partial to full GR agonist with a relatively high affinity for the GR on endogenous genes in other cells and via synthetic reporter genes [36], [41], [44], [74]. The findings of the present study suggest that in the context of the genital mucosa, these GR-mediated effects of MPA in cervical epithelial cells are likely to play a critical role in discriminating between the effects on inflammation caused by different progestins and progesterone and hence susceptibility to genital infections, given the predominant expression of the GR and lack or PR protein expression in these cells. The GR knockdown results furthermore suggest that changes in GR expression levels are likely to significantly modulate the inflammatory response in the endocervix, with reduced GR levels even possibly resulting in some pro-inflammatory effects by both MPA, NET-A and P4.

Our findings that MPA has anti-inflammatory gene expression effects in the endocervical cells are consistent with previous reports that show MPA suppresses pro-inflammatory immune markers in primary mouse uterine and cervical tissue and in primary human vaginal mucosal mononuclear cells [49], [75]. Given the different steroid receptor selectivities of MPA, NET and P4 [36]–[38], it is likely, however, that the steroid receptor profile of different compartments of the female genital tract will determine the outcome of inflammatory gene expression effects of these ligands. In the current paper we show that both primary endocervical cells and the endocervical cell line express predominantly the GR. In contrast, we have previously shown that the Ect1/E6E7 ectocervical epithelial and Vk2/E6E7 vaginal epithelial cell lines appear to express a greater variety of steroid receptors, including the PR, GR, AR and ERα [76]. This is consistent with the report that the ectocervix is covered by a mucosal layer that is histologically similar to the vagina but different to the endocervix [77]. Our previous results show that IL-6, IL-8 and RANTES mRNA levels are regulated differently in the ectocervical and vaginal cell lines compared to the endocervical cell line, consistent with their different steroid receptor profiles. MPA and NET have no effect on IL-6 mRNA levels in both the ectocervical and vaginal cell lines, while MPA is pro-inflammatory for IL-8 in the ectocervical cell line, in contrast to the anti-inflammatory results we observed for MPA in the endocervical cell line. P4 appears to be pro-inflammatory at 1 µM concentrations for most of the pro-inflammatory genes in the ectocervical and vaginal cell lines, an effect which we also observe for some genes at 1 µM P4 for IL-6, but not IL-8 or RANTES. Interestingly MPA represses RANTES in both the ectocervical and endocervical cell lines, with no significant effect in vaginal cells. However, in the ectocervical cells, this effect is mediated predominantly via the AR, while in the endocervical cells, we show that it is mediated via the GR. Interestingly, we have recently found that MPA, unlike P4 and NET, shows a very similar pattern and potency of repression of pro-inflammatory genes in human PBMCs to that observed in the current study in the endocervical cell line, with a similar predominantly GR steroid receptor profile [78]. Collectively, our results support the hypothesis that MPA, when acting predominantly via the GR, is likely to exert anti-inflammatory effects on gene expression via classical transrepression mechanisms, unlike NET and P4, but when the steroid receptor profile is changed, the responses are likely to vary. Furthermore the results collectively suggest that different compartments of the genital tract are likely to exhibit different inflammatory responses to MPA vs NET vs P4, with their associated different effects on susceptibility to genital infections. Our lack of detection of PR expression in the endocervical primary cells or cell lines raises the question as to what is the role of the PR in mediating responses to progestins and progesterone in the endocervix. It is possible that other cells besides epithelial cells in the cervix express functional PR, as is suggested from one report [79], while others report the expression of both the GR and ERα [80].

Whether the observed effects of MPA, NET and P4 are relevant to the physiological doses of these ligands *in vivo* is a critical question, which we investigated here by dose response analysis [81] to determine potencies (EC_50_s) and efficacies (maximal response). The MPA serum concentrations of DMPA-users are reported to be in the range 2.5 to 65 nM a few days after injection and to plateau at about 2.6 nM for about three months thereafter [38], [48], [82], while serum concentrations for injectable NET-EN, in the range of 1.5–59 nM have been reported [83]. The concentration of endogenous P4 in serum of premenopausal women is low during the follicular phase (0.65 nM) but rises to about 80 nM during the luteal phase, and to about 600 nM during pregnancy [37]. We show that MPA at 10 nM significantly represses both IL-6 and RANTES at 24 hrs (Figure 3 C and H). Furthermore our dose response analysis show that MPA has a potency of ∼24 nM for transactivation of the anti-inflammatory GILZ gene and a potency of ∼4–20 nM for repression of the pro-inflammatory IL-6, IL-8 and RANTES genes. This suggests that these immunosuppressive effects are likely to be relevant at physiological doses of MPA used in injectable contraception, particularly shortly after injection, while any possible effects of NET-EN injectable contraceptive on inflammation via the GR are likely to be negligible, even shortly after injection. Since P4 at concentrations up to 100 nM shows very little effect on expression of the genes investigated, P4 at doses other than during pregnancy, are unlikely to exert major effects on inflammation or immune function in endocervical epithelial cells. However, about 20% repression of IL-6 is observed by P4 at 4 hrs at 10–100 nM, suggesting that in the presence of a predominant GR, P4 could exert some anti-inflammatory effects. At pregnancy concentrations, P4 may exert some pro-inflammatory effects on some genes, as suggested by our dose response analysis showing this trend for some genes at 1 µM. It should, however, be noted that the concentrations of MPA, NET and P4 in cervical tissue may not be the same as that found in the serum of contraceptive users.

The physiological significance of changes in expression of pro-inflammatory mediators like IL-6, IL-8 and RANTES in genital epithelial cells is difficult to predict. Increased pro-inflammatory mediators could increase recruitment of dendritic cells (DCs) or Langerhans cells (LCs) as well as CD4^+^ T cells and monocytes/macrophages, thus potentially increasing HIV-1 acquisition by increasing the number of target cells. Thus progestins like MPA, unlike NET-A, that exert anti-inflammatory gene expression effects in the female genital tract may decrease HIV-1 acquisition by decreasing the number of target cells. However, decreased pro-inflammatory mediators could also inhibit immune function, such as B-cell maturation, T-cell activation and differentiation, IgA production, neutrophil/monocyte/macrophage/dendritic cell activity, reducing the host’s ability to mount a defence against a pathogen [84]. Additionally, RANTES is a ligand for the CCR5 receptor, which has the ability to block HIV-1 entry [85]. Thus a decrease in RANTES expression by endocervical epithelial cells, as we show for MPA but not NET, with a potency of 5 nM, could increase HIV-1 infection of CD4^+^ T cells*in vivo.* Interestingly, MPA shows the greatest efficacy for RANTES mRNA repression, acting like a full GR agonist. Whether or not DMPA usage increases or decreases the expression of inflammatory mediators in the female genital tract *in vivo* is unclear and requires further investigation. Several studies suggest that a pro-inflammatory environment is associated with an increase in HIV-1 acquisition [86]–[88]. Furthermore, DMPA-usage was recently reported to be associated with increased HIV-1 acquisition and increased levels of RANTES [88]. However, in this study it was not possible to discriminate between elevated RANTES levels being a cause of infection or a consequence of exposure to HIV-1 prior to seroconversion. Additionally, since 76% of the DMPA users in this study were positive for an STI, it may be that elevated RANTES was a consequence of STI infection, despite attempts to correct for that confounding variable. In contrast to the latter study, the study by Huijbregts et al. found that DMPA-usage is associated with immunosuppressive effects in the cervical mucosa [49]. Furthermore, we have recently shown that MPA, unlike NET or P4, increases apoptosis of T-cells, which is potentiated after HIV-1 infection [74], potentially decreasing the ability of T-cells to mount an anti-viral defence. Currently available clinical data from women on DMPA, taken together with animal data plus our and other biochemical *ex vivo* data, certainly suggest that immunosuppressive properties of long term MPA contraceptive usage may be a significant factor contributing towards increasing HIV-1 acquisition, transmission and possibly disease progression. Importantly, our results show that MPA effects on genital mucosal immune function and susceptibility to infections are likely to be very different to those of NET and P4, when mediated via the GR, and that choice and concentration of progestin in contraception are likely to be critical factors.

## Supporting Information

Figure S1 **Only DEX and MPA represses basal as well as TNF-induced cytokine mRNA expression.** End1/E6E7 cells were treated for 24 hrs with 100 nM DEX, MPA, P4, NET-A or vehicle (ethanol) (CTRL), in the absence or presence of 20 ng/ml TNFα. Thereafter the cells were harvested, total RNA was isolated and reverse-transcribed. Relative (A) IL-6, (B) IL-8 and (C) RANTES mRNA expression was measured by real-time qRT-PCR and normalised to GAPDH mRNA expression. In addition, relative gene expression was normalized to basal activity (CTRL) in order to obtain relative fold expression. Graph represents pooled results of at least three independent experiments and are plotted as mean ± SEM. Statistical analysis was carried out using GraphPad Prism software (version 5) using a one-way ANOVA with Dunnett post-test, followed by a student’s t-test to compare specific conditions to each other. Statistical significance is denoted by * or ** to indicate P<0.05 or P<0.001, respectively.
(TIF)

Figure S2 **DEX, MPA and NET-A induce total IκBα protein.** End1/E6E7 cells were treated for 24 hrs with 100 nM DEX, MPA, P4, NET-A or vehicle (ethanol) (CTRL). Thereafter, cells were harvested and equal volumes of lysate were analysed by **(A)** Western blotting with an antibody specific for total IκBα and a GAPDH specific antibody as loading control. **(B)** Western blots of five independent experiments were quantified to determine the relative GR protein expression. Statistical analysis was carried out using GraphPad Prism software (version 5) using a one-way ANOVA with a Dunnett post-test followed by a student’s t-test to compare specific conditions to each other. Statistical significance is denoted by *, ** or *** to indicate P<0.05, P<0.001 or P<0.0001, respectively.
(TIF)

Figure S3 **Cell Viability of VEN-100.** VEN-100 cells were either incubated for 24 hrs (day 1, treatment day) or 72 hrs (day 3, end of treatment day), followed by, analysis for cell viability (MTT assay). Absorbance readings were measured at 570 nm. Cell culture media served as the control (CTRL). CTRL for each day was set to 1 to obtain relative fold cell viability. The graph represents results of at least three independent experiments, plotted mean +/− SEM. Statistical analysis was carried out using GraphPad Prism software (version 5) using a one-way ANOVA with a Dunnett post-test followed by a student’s t-test to compare specific conditions to each other. Statistical significance is denoted by ** or *** to indicate P<0.001 or P<0.0001, respectively.
(TIF)

Figure S4 **Ligand-selective GR protein turnover.** End1/E6E7 cells were treated with increasing amounts (1 nM, 10 nM, 100 nM and 1 µM) of DEX, MPA, P4 or NET-A, or vehicle (ethanol) (CTRL) for 24 hrs. Thereafter, the cells were harvested and equal volumes of lysate were analysed by Western blotting with antibodies specific for GR and GAPDH as loading control.
(TIF)
