# Supplementary figures and images for: The Injectable-Only Contraceptive Medroxyprogesterone Acetate, Unlike Norethisterone Acetate and Progesterone, Regulates Inflammatory Genes in Endocervical Cells via the Glucocorticoid Receptor
Source: PLoS One. 2014 May 19;9(5):e96497. doi: 10.1371/journal.pone.0096497 (PMC4026143; doi:10.1371/journal.pone.0096497)

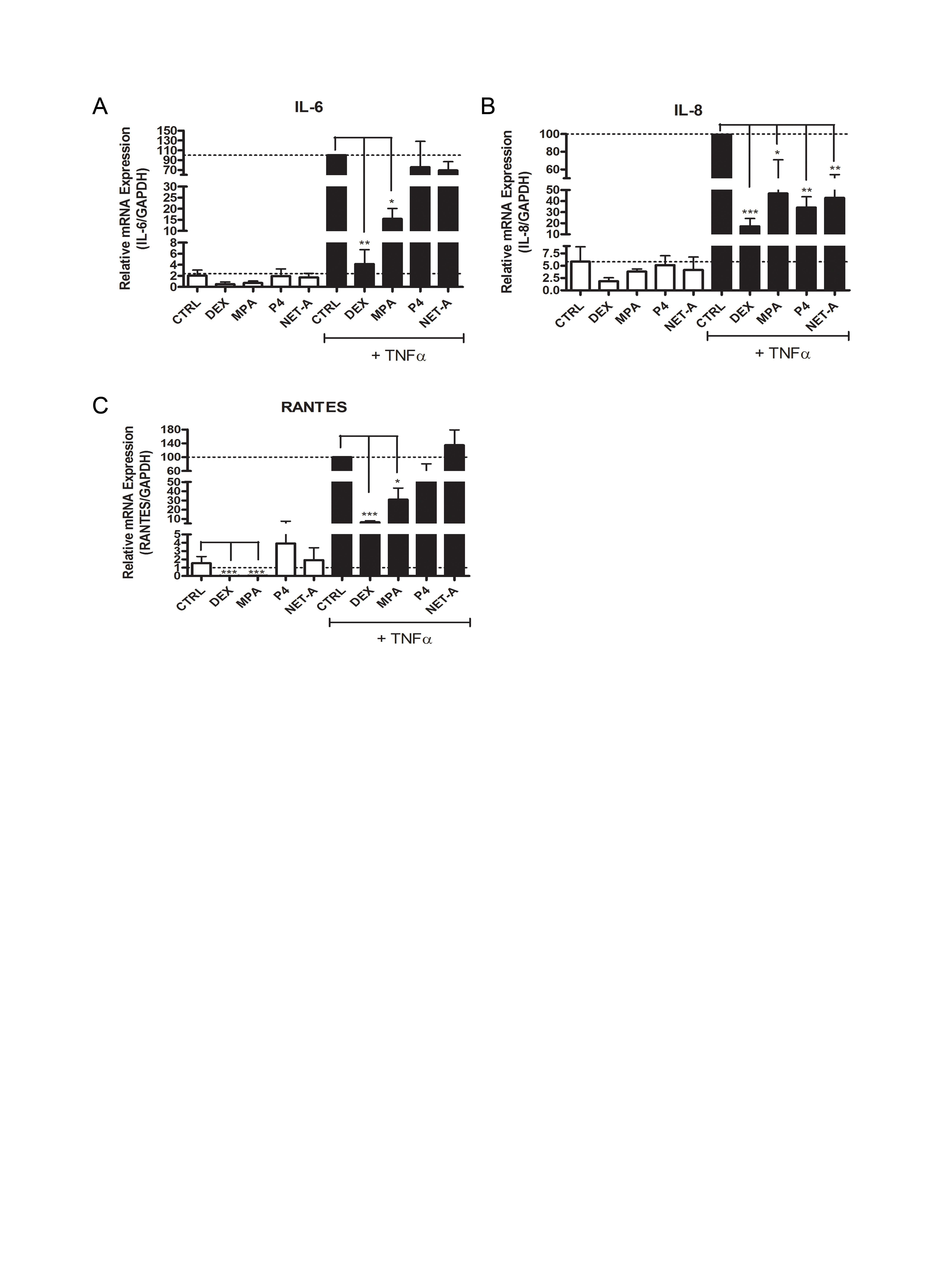

Supplement: Figure S1 — Only DEX and MPA represses basal as well as TNF-induced cytokine mRNA expression. End1/E6E7 cells were treated for 24 hrs with 100 nM DEX, MPA, P4, NET-A or vehicle (ethanol) (CTRL), in the absence or presence of 20 ng/ml TNFα. Thereafter the cells were harvested, total RNA was isolated and reverse-transcribed. Relative (A) IL-6, (B) IL-8 and (C) RANTES mRNA expression was measured by real-time qRT-PCR and normalised to GAPDH mRNA expression. In addition, relative gene expression was normalized to basal activity (CTRL) in order to obtain relative fold expression. Graph represents pooled results of at least three independent experiments and are plotted as mean ± SEM. Statistical analysis was carried out using GraphPad Prism software (version 5) using a one-way ANOVA with Dunnett post-test, followed by a student’s t-test to compare specific conditions to each other. Statistical significance is denoted by * or ** to indicate P<0.05 or P<0.001, respectively. (TIF) [file pone.0096497.s001.tif]

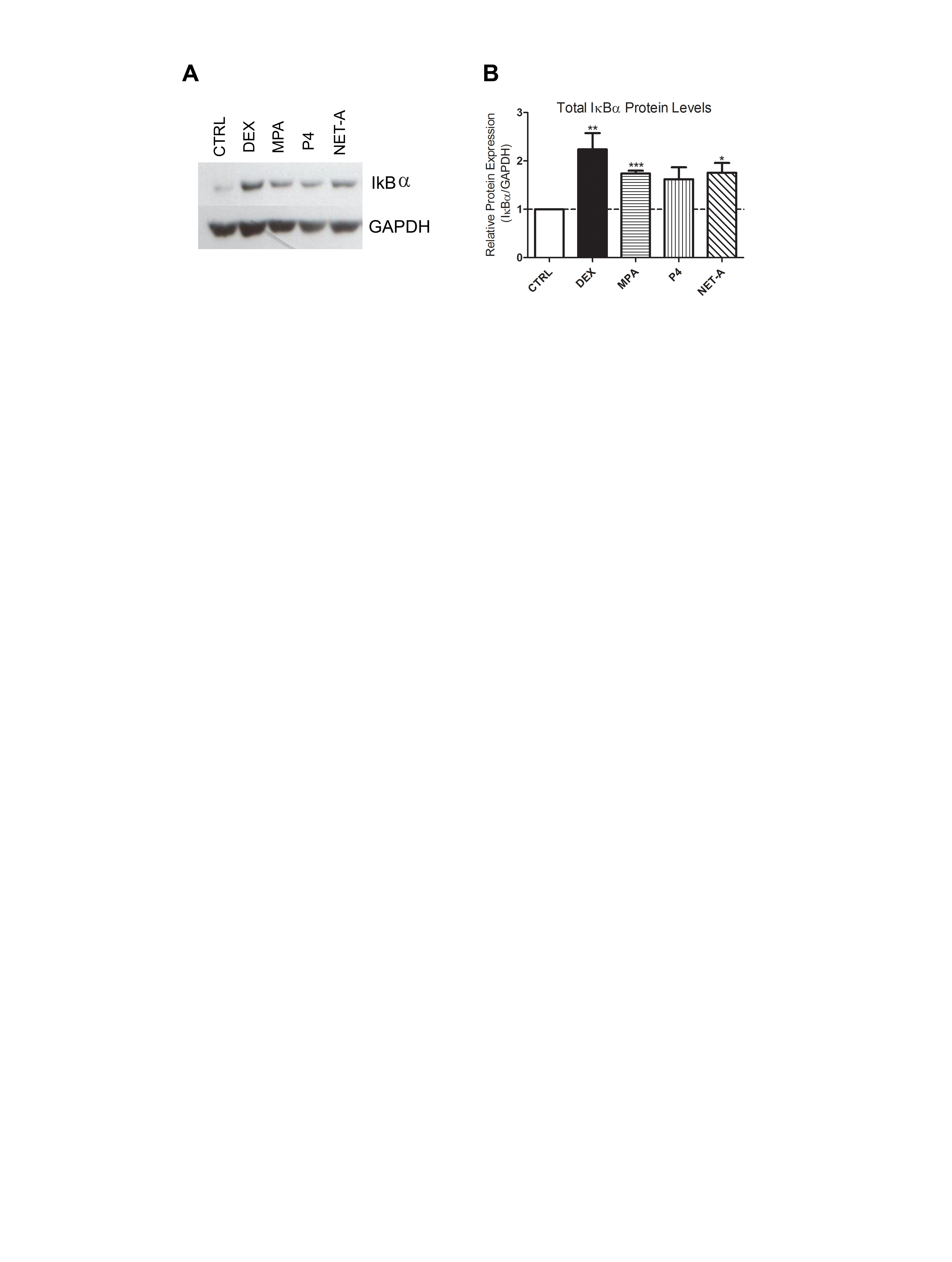

Supplement: Figure S2 — DEX, MPA and NET-A induce total IκBα protein. End1/E6E7 cells were treated for 24 hrs with 100 nM DEX, MPA, P4, NET-A or vehicle (ethanol) (CTRL). Thereafter, cells were harvested and equal volumes of lysate were analysed by (A) Western blotting with an antibody specific for total IκBα and a GAPDH specific antibody as loading control. (B) Western blots of five independent experiments were quantified to determine the relative GR protein expression. Statistical analysis was carried out using GraphPad Prism software (version 5) using a one-way ANOVA with a Dunnett post-test followed by a student’s t-test to compare specific conditions to each other. Statistical significance is denoted by *, ** or *** to indicate P<0.05, P<0.001 or P<0.0001, respectively. (TIF) [file pone.0096497.s002.tif]

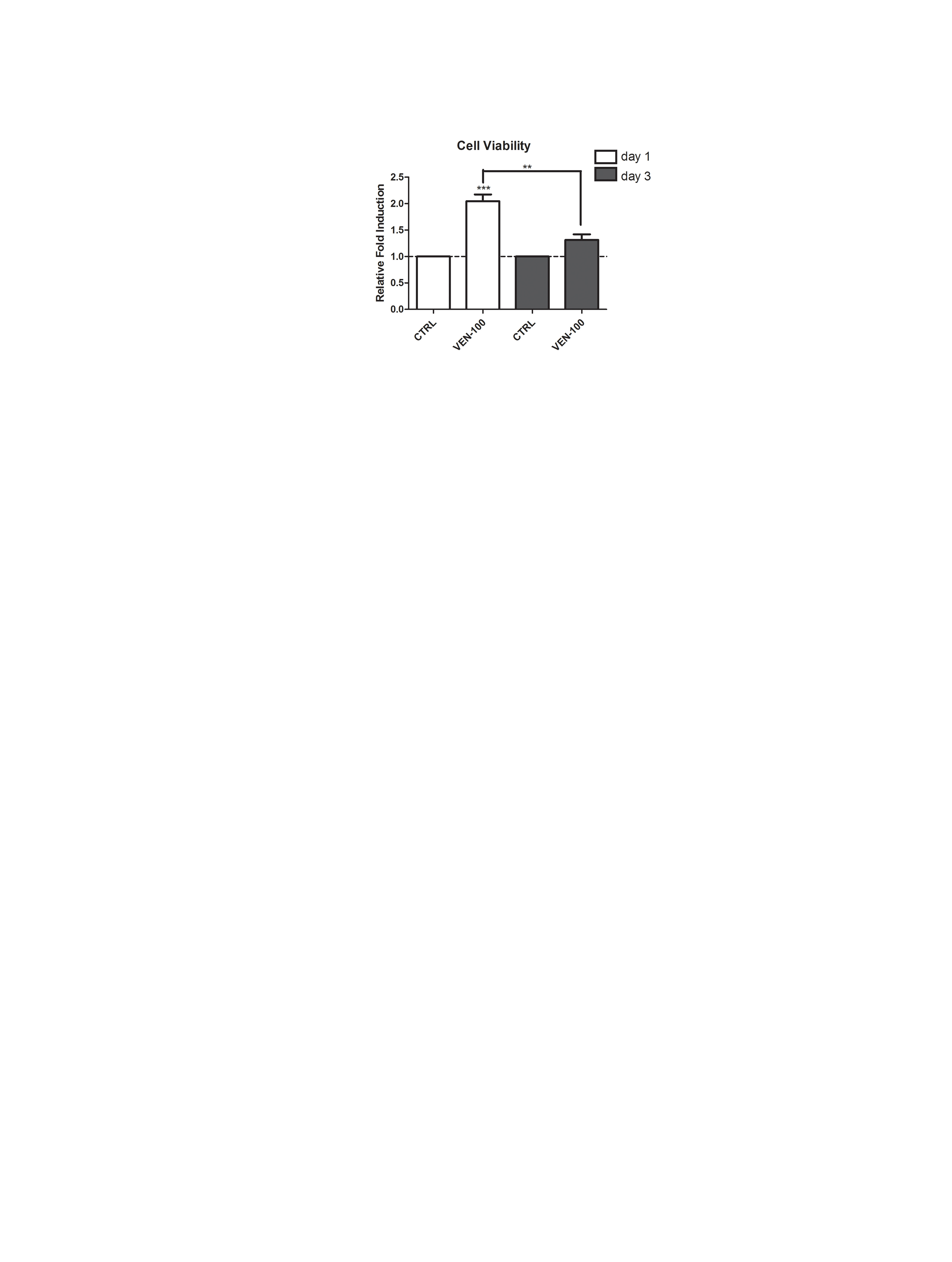

Supplement: Figure S3 — Cell Viability of VEN-100. VEN-100 cells were either incubated for 24 hrs (day 1, treatment day) or 72 hrs (day 3, end of treatment day), followed by, analysis for cell viability (MTT assay). Absorbance readings were measured at 570 nm. Cell culture media served as the control (CTRL). CTRL for each day was set to 1 to obtain relative fold cell viability. The graph represents results of at least three independent experiments, plotted mean +/− SEM. Statistical analysis was carried out using GraphPad Prism software (version 5) using a one-way ANOVA with a Dunnett post-test followed by a student’s t-test to compare specific conditions to each other. Statistical significance is denoted by ** or *** to indicate P<0.001 or P<0.0001, respectively. (TIF) [file pone.0096497.s003.tif]

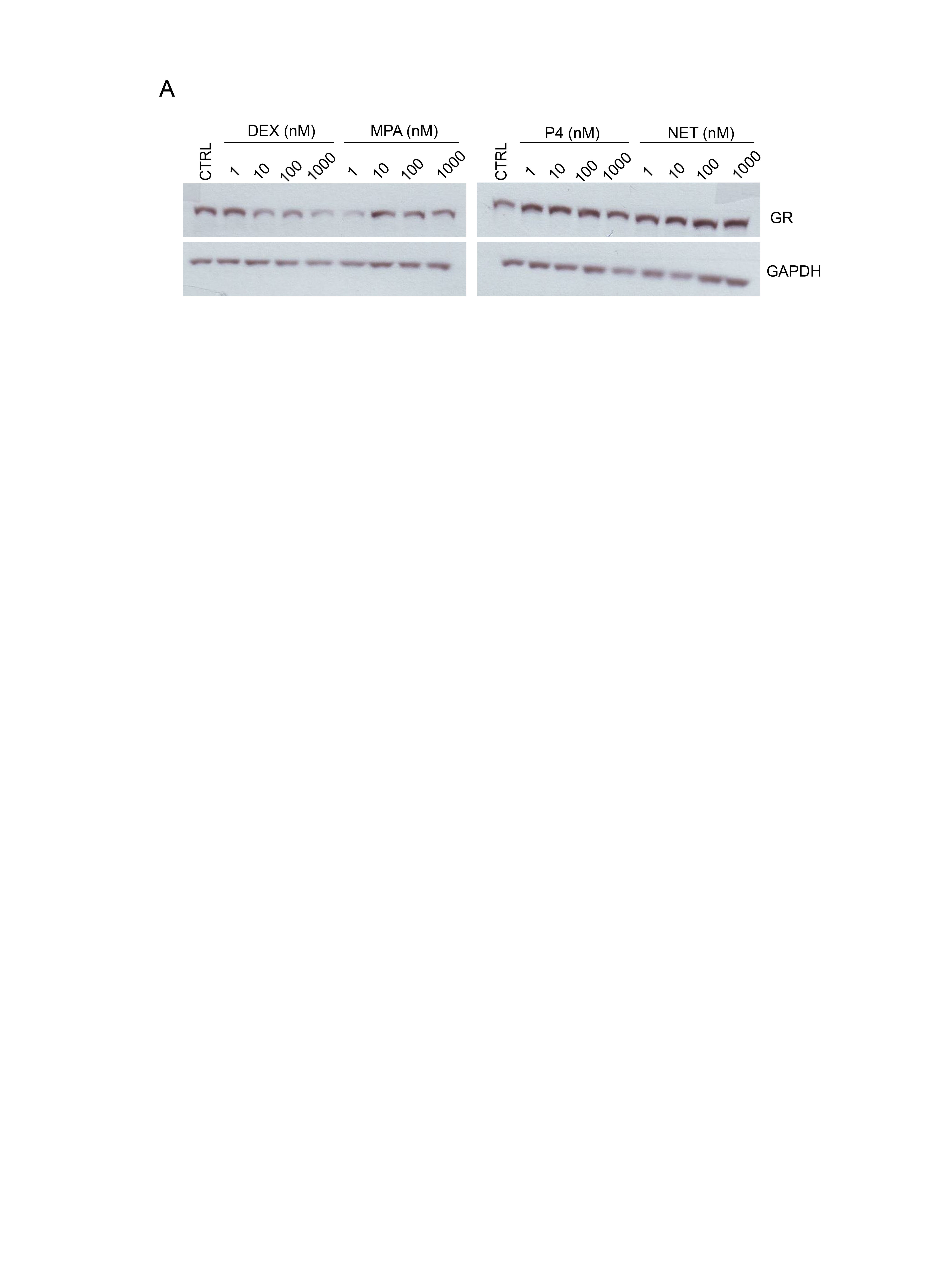

Supplement: Figure S4 — Ligand-selective GR protein turnover. End1/E6E7 cells were treated with increasing amounts (1 nM, 10 nM, 100 nM and 1 µM) of DEX, MPA, P4 or NET-A, or vehicle (ethanol) (CTRL) for 24 hrs. Thereafter, the cells were harvested and equal volumes of lysate were analysed by Western blotting with antibodies specific for GR and GAPDH as loading control. (TIF) [file pone.0096497.s004.tif]
